# A case of acute generalized exanthematous pustulosis following administration of nemolizumab

**DOI:** 10.1016/j.jdcr.2025.04.033

**Published:** 2025-05-21

**Authors:** Alyssa Khoo, Elgida Volpicelli, Elle de Moll

**Affiliations:** a Department of Dermatopathology, Stamford Hospital, Stamford, Connecticut; b Department of Dermatology, Yale School of Medicine, New Haven, Connecticut

**Keywords:** acute generalized exanthematous pustulosis, biologics, drug eruption, nemolizumab

## Introduction

Acute generalized exanthematous pustulosis (AGEP) is a cutaneous drug reaction manifested as coalescing plaques of tiny pustules, most commonly caused by systemic medication or viral infection.^1^ AGEP is a rare condition, affecting approximately 1 to 5 people per million annually.^2^ Nemolizumab is an antibody that targets interleukin receptor interleukin 31 receptor A and was recently US Food and Drug Administration-approved to treat patients with prurigo nodularis and atopic dermatitis (AD).^3^ Nemolizumab suppresses the skin’s T helper 2 and interleukin 4/interleukin 13 immune responses, which can significantly lessen itch intensity.^4^ Transcriptomic alterations driven by nemolizumab are also correlated with the healing of skin lesions, regulation of extracellular matrix remodeling, and maintenance of cutaneous nerve functioning.^5^ The most common side effects occurring in patients on nemolizumab are injection site reactions, exacerbation of AD, and headache.^6^ We present a case of a 76-year-old man experiencing AGEP after administration of nemolizumab for AD.

## Case report

A 76-year-old man presented with adult-onset eczema. He had previously failed narrow band UV-B with no relief of itch after approximately 10 sessions. He was treated with triamcinolone 0.1% cream, tofacitinib 2% cream, calcipotriene 0.005% cream, fexofenadine 180 mg twice daily, and hydroxyzine 25 mg nightly, with minimal benefit. In July 2024, the patient reported poor quality of life due to persistent itching, and he received 60 mg of intramuscular kenalog and fluocinonide 0.05% cream. His itch continued to worsen, and he was started on methylprednisolone taper, which helped significantly. A biopsy of the anterior aspect of the left thigh showed sparse perivascular dermatitis with pigment-laden macrophages, and the anterior aspect of the right thigh, showed perivascular and interstitial dermatitis, with focal epidermal necrosis and mixed cells including eosinophils. Eczema was the favored clinical diagnosis despite an equivocal biopsy given the clinical presentation of clear eczematous plaques and significant pruritus.

The decision was made to proceed with dupilumab given the severity of symptoms. Seven days after this loading dose, the patient experienced a flare of worsening itch, which was managed with methylprednisolone by his primary care physician. Additionally, the patient experienced worsening of his ocular rosacea, with eye dryness and irritation, which was treated with steroid eye drops.

**Fig 1:**
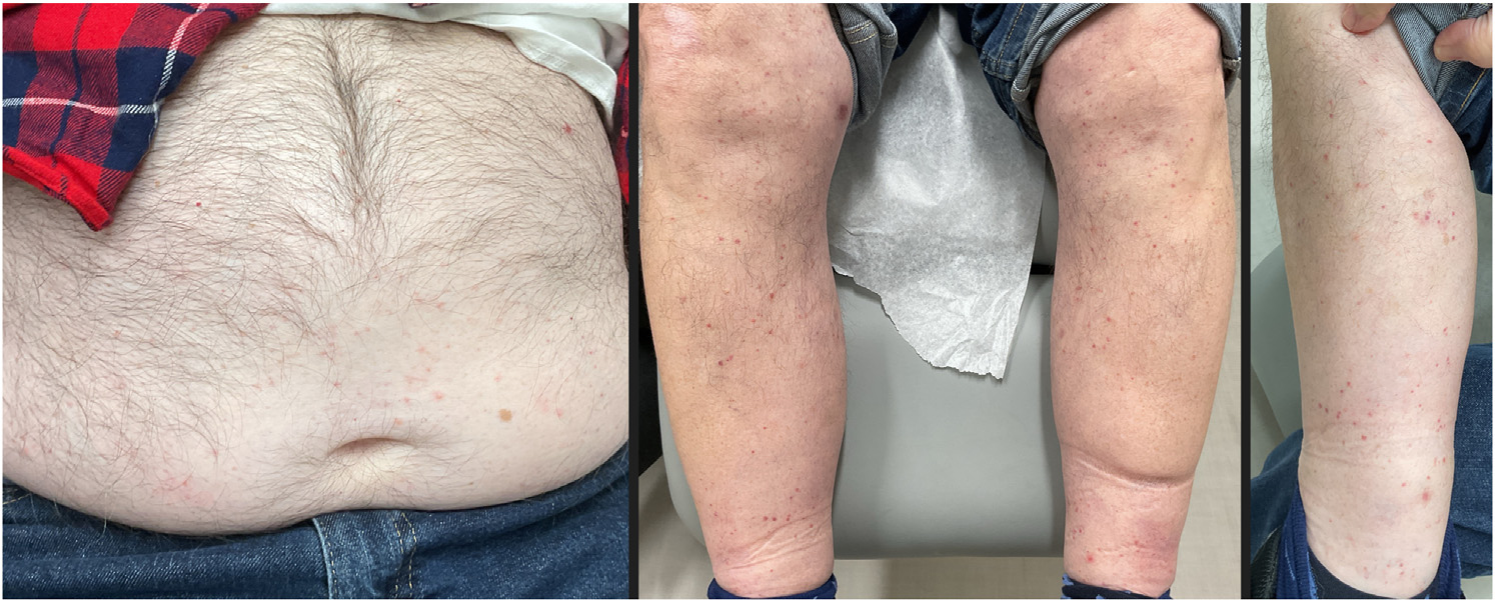
Diffuse erythematous papules on the legs and abdomen, after the initial nemolizumab dose.

Given the discomfort of his ocular symptoms, dupilumab was discontinued after receiving the loading dose. The patient tried various topicals for another 3 months before deciding to pursue nemolizumab. Within 2 days of receiving nemolizumab (60 mg), the patient reported near-complete resolution of his pruritus. However, 5 days later, the patient experienced a new rash with small erythematous papules which progressed into pustules, predominantly on the abdomen, legs, and arms (Fig 1). The patient denied any pruritus or any new exposures.

**Fig 2:**
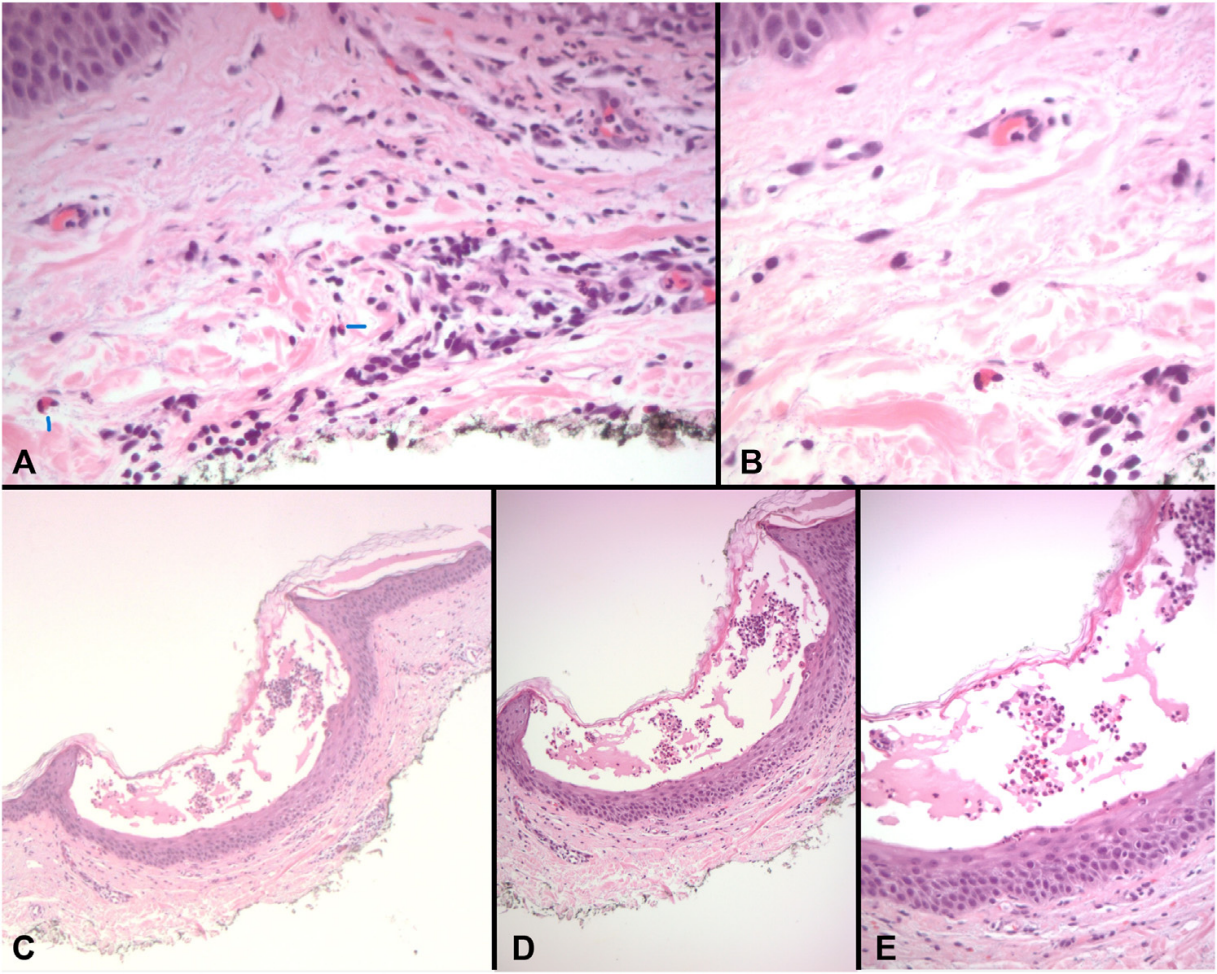
Histopathology of a biopsy from the left superior quadrant of the abdomen with subcorneal pustular dermatosis. **A,** Scattered dermal eosinophils marked with blue lines, (**B**) dermal eosinophil, (**C**) subcorneal neutrophilic pustule, (**D**) Subcorneal neutrophil pustule, and (**E**) subcorneal neutrophilic pustule. Eosinophils are also present. (Original magnifications: **A** and **E,** ×200; **B,** ×400; **C,** ×40; **D,** ×100.)

The presentation of AGEP was subtle, prompting biopsy as well as consideration of viral and antibiotic etiologies. The pustules were smaller and more discrete than typically seen in AGEP, but the pathologic features were diagnostic of AGEP. The histologic sections demonstrated subcorneal clefting with a collection of neutrophils in the cavity. There was a superficial perivascular mixed inflammatory infiltrate (Fig 2). AGEP was treated with topical triamcinolone, as it seemed to be a particularly mild case, and was responsive over the subsequent 2 weeks.

The patient returned to the clinic 13 days after the initial nemolizumab dose, appearing with skin cleared of the drug eruption. At the time, the patient remembered that he had been started on doxycycline for an unrelated tooth abscess before starting nemolizumab. Given the concurrent antibiotics, it was unclear if AGEP was secondary to doxycycline or nemolizumab. After a long conversation about risks and benefits with the patient, the decision was made to redose nemolizumab at 4 weeks as scheduled.

Within 4 days of receiving the 30 mg of nemolizumab, an identical drug rash recurred diffusely, linking AGEP to nemolizumab. The patient responded again to topical triamcinolone after the redose.

AGEP was not reported in the clinical trials and has not yet been reported in the literature in association with nemolizumab.

## Discussion

Nemolizumab monotherapy was US Food and Drug Administration-approved for prurigo nodularis and AD in 2024 in the United States. During clinical trials, the most common dermatologic side effect was eczema. Masuyuki et al^7^ reported a case of a 62-year-old man patient experiencing bullous pemphigoid after a single administration of nemolizumab for prurigo-type AD.^8^ According to our review of the preexisting literature, our case study is the first to report nemolizumab causing AGEP.

AGEP is most frequently caused by antibiotics, specifically those beta-lactams, including aminopenicillins and cephalosporins. Other medications including hydroxychloroquine, ketoconazole, fluconazole, and terbinafine have also been established as AGEP triggers.^8^

Biologic medications are less commonly associated with AGEP, but rare cases have been reported. Masood et al^9^ presented a 66-year-old man patient who experienced biopsy-proven AGEP from cetuximab treatment for squamous cell carcinoma. Additionally, Wu et al^10^ described a case of a 17-year-old girl patient with AD who experienced AGEP after a single injection of dupilumab.^10^

Biologic medications are relatively novel therapies, with limited long-term efficacy data, whether used alone or in combination with other biologics, topical corticosteroids, phototherapy, or other immunosuppressive agents. Our case emphasizes the importance of ongoing vigilance to identify and manage less common adverse effects associated with dermatological biologics that are not always identified in clinical trials.

## Conflicts of interest

None disclosed.
